# Glycaemia and ischaemia-reperfusion brain injury in patients with ischaemic stroke treated with mechanical thrombectomy (GLIAS-MT): an observational, unicentric, prospective study protocol

**DOI:** 10.1136/bmjopen-2024-086745

**Published:** 2024-08-07

**Authors:** Carlos Hervás, Irene Peirotén, Laura González, María Alonso de Leciñana, Elisa Alonso-López, Laura Casado, Elena De Celis-Ruíz, Andrés Francisco Fernández Prieto, Remedios Frutos, Rebeca Gallego-Ruiz, Noemí González Pérez de Villar, María Gutiérrez-Fernández, Pedro Navia, Laura Otero-Ortega, Javier Pozo-Novoa, Ricardo Rigual, Jorge Rodríguez-Pardo, Gerardo Ruiz, Blanca Fuentes

**Affiliations:** 1 Department of Neurology and Stroke Centre, La Paz University Hospital and Department of Medicine, Universidad Autónoma de Madrid, La Paz University Hospital Research Institute (IdiPAZ), Madrid, Spain; 2 Department of Radiology (Neurointerventional Radiology), La Paz University Hospital, La Paz University Hospital Research Institute (IdiPAZ), Madrid, Spain; 3 Neurological Sciences and Cerebrovascular Research Laboratory, La Paz University Hospital Research Institute (IdiPAZ), Madrid, Spain; 4 Diabetes Unit, Department of Endocrinology, La Paz University Hospital, La Paz University Hospital Research Institute (IdiPAZ), Madrid, Spain

**Keywords:** Stroke, NEUROLOGY, Neuroradiology, General diabetes

## Abstract

**Introduction:**

Poststroke hyperglycaemia is an independent risk factor for poorer outcomes in patients treated with mechanical thrombectomy (MT) and is associated with a lower probability of functional recovery and higher mortality at 3 months. This study aims to evaluate the association between glucose levels during cerebral reperfusion with MT and functional recovery at 3 months, measured by subcutaneous continuous glucose monitoring (CGM) devices.

**Methods:**

This prospective observational study aims to recruit 100 patients with ischaemic stroke and large anterior circulation vessel occlusion, in whom MT is indicated. CGM will be performed using a Freestyle Libre ProIQ device (FSL-CGM, Abbott Diabetes Care, Alameda, California, USA), which will be implanted on admission to the emergency department, to monitor glucose levels before, during and after reperfusion. The study’s primary endpoint will be the functional status at 3 months, as measured by the dichotomised modified Rankin Scale (0–2 indicating good recovery and 3–6 indicating dependency or death). We will analyse expression profiles of microRNA (miRNA) at the time of reperfusion and 24 hours later, as potential biomarkers of ischaemic-reperfusion injury. The most promising miRNAs include miR-100, miR-29b, miR-339, miR-15a and miR-424. All patients will undergo treatment according to current international recommendations and local protocols for the treatment of stroke, including intravenous thrombolysis if indicated.

**Ethics and dissemination:**

This study (protocol V.1.1, dated 29 October 2021, code 6017) has been approved by the Clinical Research Ethics Committee of La Paz University Hospital (Madrid, Spain) and has been registered in ClinicalTrials.gov (NCT 05871502). Study results will be disseminated through peer-reviewed publications in Open Access format and at conference presentations.

**Trial registration number:**

https://www.clinicaltrials.govNCT05871502.

**STRENGTHS AND LIMITATIONS OF THIS STUDY**

- The GLIAS-mechanical thrombectomy (MT) is a prospective observational study that builds on previous research into the prognostic impact of hyperglycaemia in stroke patients treated with MT.
- Continuous glucose monitoring devices present a valuable opportunity to monitor glucose levels during MT and to investigate their impact at the time of reperfusion on the ischaemia-reperfusion damage.
- The observational nature of this study entails the possibility of biases.
- The fact that patients will be recruited in a single centre and the small sample size could be limitations for external validity.

## Introduction

 Poststroke hyperglycaemia is a very common complication in ischaemic stroke, affecting approximately two-thirds of patients, and is associated with poorer recanalisation rates, reduced perfusion, increased damage due to ischaemia-reperfusion and, consequently, poorer patient outcomes.^1 2^ Furthermore, hyperglycaemia can counteract the potential therapeutic effect of reperfusion treatments such as intravenous thrombolysis^3 4^ and mechanical thrombectomy (MT).^5^,^8^

Hyperglycaemia can cause damage through several pathophysiological mechanisms, including abnormalities in the blood–brain barrier and increased lactic acid production.^1 2^ Poststroke hyperglycaemia has been independently associated with poor outcomes after MT, with lower rates of functional recovery,^6 7 9^ a higher risk of haemorrhagic transformation^7 9 10^ and cerebral oedema^11 12^ and increased mortality at 3 months.^7^ The GLycemia in Acute Stroke (GLIAS) study was the first multicentre study to establish prognostic threshold for hyperglycaemia at 155 mg/dL, demonstrating a correlation between elevated glucose levels in the acute phase of stroke and poor outcomes, independently of stroke severity, infarct volume, diabetes or age.^13^

In patients treated with MT, the Solitaire Flow Restoration With the Intention for Thrombectomy (SWIFT) clinical trial showed that for each 10 mg/dL increase in blood glucose values, there was a 42% reduction in the probability of an excellent recovery at 3 months.^6^ It has yet to be determined whether administering insulin therapy prior to and during MT can mitigate the negative impact of hyperglycaemia in stroke recovery. Several studies have attempted to analyse this impact based on retrospective discrete determinations, such as glycaemia on admission^5 7 8 14^ and 24–48 hours after stroke,^15^,^17^ but none of them have monitored glycaemia during the MT procedure.

Despite the mounting evidence of its deleterious effects, poststroke hyperglycaemia is currently undertreated, which could be due to the lack of specific recommendations.^18^,^20^ The Highly Effective Reperfusion using Multiple Endovascular deviceS (HERMES) collaboration highlighted the significant variation in glycaemic management guidelines during the acute phase of stroke across various MT clinical trials: four of the trials had no specific recommendations, and one advised against hyperglycaemia treatment.^5^ Periprocedural management primarily focuses on blood pressure optimisation and selection of anaesthetic technique while glucose-level control is usually relegated to the postprocedural phase.^21 22^ In daily clinical practice, glucose levels are not monitored during the procedure, despite being one of the main modifiable prognostic factors in the acute phase of ischaemic stroke and closely related to reperfusion damage. Determining the impact of poststroke hyperglycaemia on patient outcomes could, therefore, lead to a paradigm shift in clinical practice.

The development and use of subcutaneous continuous glucose monitoring (CGM) devices^23 24^ provides an excellent opportunity to investigate the prognostic impact of poststroke hyperglycaemia in patients with acute stroke. CGM devices are minimally invasive, measure glucose levels in the interstitial fluid every 5 min and have been shown to be safe in patients with stroke during research studies,^25^,^29^ including those treated with intravenous thrombolysis^26^ and, more recently, in patients who underwent endovascular therapy.^30^ The feasibility, diagnostic accuracy and safety of CGM in the anaesthetic setting have also been demonstrated.^31^

Few studies have analysed the effect of hyperglycaemia after stroke on the levels of biomarkers of brain damage and repair.^32^ Recently, a number of micro-RNAs (miRNAs) have been proposed to modify the response to ischaemia-reperfusion injury and to regulate the expression of several elements essential for cell survival and apoptosis. Consequently, there is a growing interest in the potential role of miRNAs as biomarkers of ischaemia-reperfusion injury and even as potential therapeutic targets.

These miRNAs are of interest due to their functions, with previous studies demonstrating a role in cerebral ischaemia. In the context of ischaemic stroke, miR-339, miR-15a, miR-424 and miR-100 have been associated with large vessel occlusion.^33 34^ MiR-339, which is overexpressed in cases of cerebral and myocardial ischaemia,^35^ is related to neuronal survival and suppression of apoptosis in ischaemic conditions.^36^ MiR-15 and miR-424 have been linked to an antiangiogenic effect through VEGF inhibition.^37^ Additionally, miR-100 has been demonstrated to have an antiatherosclerotic effect.^38^ Furthermore, this miRNA exhibits varying levels in patients with cerebral infarction due to large vessel occlusion, suggesting a potential association with functional recovery.^33^ Finally, miR-29b has been demonstrated to attenuate ischaemic injury by negatively regulating the p53-dependent apoptosis pathway and could, therefore, be a potential target in diminishing cell injury in ischaemic stroke.^39^

We hypothesised that those patients with glycaemia values <155 mg/dL during MT and especially at the time of reperfusion will have less ischaemia-reperfusion injury, showing a different miRNA expression profile, with better neurological and functional outcomes and a lower risk of haemorrhagic transformation and cerebral oedema.

## Methods and analysis

### Design

#### Clinical study

Observational, unicentric, prospective study.

#### Patient population

The inclusion criteria are as follows: male and female patients older than 18 years, ischaemic stroke with anterior circulation large-vessel occlusion confirmed by imaging, indication of MT, previous modified Rankin Scale (mRS) 0–1, signed informed consent and enrolment prior to the MT procedure. Exclusion criteria are current drug or alcohol use dependence, imaging evidence of posterior circulation occlusion, comorbidity that prevents follow-up for 3 months after stroke or participation in another trial (table 1).

**Table 1:** Inclusion and exclusion criteria

Inclusion criteria	Exclusion criteria
Male and female patients older than 18 years	Current drug or alcohol use dependence
mRS score before stroke of 0–1	Serious or life-threatening comorbidity that prevents follow-up for 3 months after the stroke
CT, angio-CT or angio-MRI evidence of stroke due to occlusion of a large vessel of the anterior circulation, including internal carotid artery (intracranial or extracranial) or middle cerebral artery (M1 or M2 segment) with indication for MT according to clinical practice	CT, angio-CT or angio-MRI evidence of posterior circulation occlusion
Informed consent signed and patient enrolment prior to the endovascular procedure	Participation in another therapeutic clinical trial

#### Randomisation

None (observational study).

#### Intervention

Patients who meet the inclusion criteria and none of the exclusion criteria will be invited to participate in this research study. The attending stroke neurologist will provide and explain the study information document to the patient (or to the patient’s family member if the patient is unable to give consent personally). This informed consent process will take place in parallel with the preparation of the MT and will not delay any therapeutic procedure. Whenever the informed consent has been signed by a family member and once the patient is able to consent, the patient will be informed about the research study and given the opportunity to reconsider or withdraw.

After signing the informed consent document and before starting the MT procedure, a subcutaneous CGM device will be placed, removing it on day 15 or at discharge, whichever comes first. CGM will be performed using a Freestyle Libre ProIQ device (FSL-CGM, Abbot Diabetes Care, Alameda, California, USA), which was selected from those currently available on the market, with previous studies documenting its safety and suitability for radiological procedures.

All patients will be managed according to current guidelines for the diagnosis and treatment of stroke, including the administration of thrombolytics if the established criteria for this treatment are met. Anaesthetic management, including the choice of general anaesthesia or conscious sedation, will follow the local protocols of the anaesthesiology department. Patients with glucose levels >155 mg/dL will receive insulin at the discretion of the attending physician, following the local protocols for managing hyperglycaemia in hospitalised patients and the guidelines for managing hyperglycaemia in patients with stroke (online supplemental table 1). Patients who underwent only angiography without MT treatment due to evidence of complete revascularisation at the time of arteriography will be excluded from the analysis.

#### Study schedule

**Figure 1:**
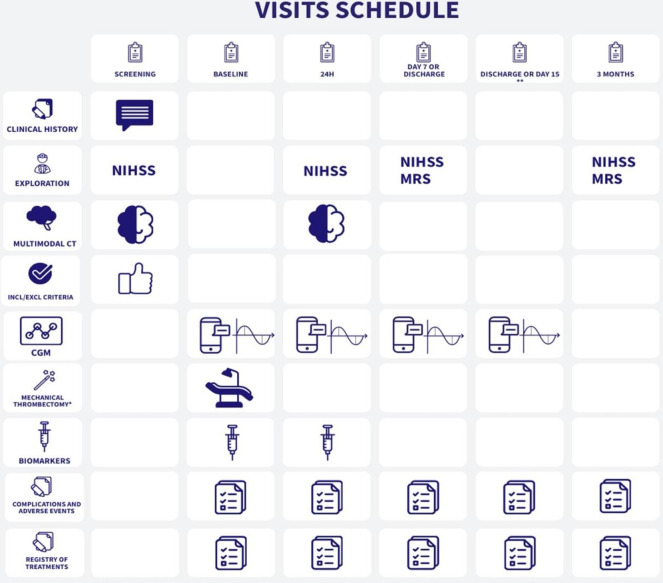
Study schedule. *Every patient fulfilling criteria for intravenous thrombolysis will be treated with it prior the performance of mechanical thrombectomy, per good clinical practice. **CGM devices will be removed at discharge or at day 15 in those patients still hospitalised. CGM, continuous glucose monitoring; mRS, modified Rankin Scale; NIHSS, National Institutes of Health Stroke Scale.

The study will last 3 months for each patient, according to the schedule illustrated in figure 1.

#### Primary endpoints

- Stroke recovery at 3 months was measured by the dichotomised mRS (0–2 indicating good functional recovery and 3–6 indicating death or dependency).

#### Secondary endpoints

- Infarct volume at 24 hours.
- The presence of any degree of haemorrhagic transformation and symptomatic haemorrhagic transformation at 24 hours.
- MicroRNA (miR-29b, miR-339, miR-15a, miR-100 and miR-424) expression profiles at the time of reperfusion and 24 hours later.
- Neurological recovery at 24 hours, using the National Institutes of Health Stroke Scale (NIHSS).
- Neurological and functional recovery at hospital discharge, using NIHSS and mRS.

#### Data collection and management

- Glucose levels at the time of reperfusion in patients achieving thrombolysis in cerebral infarction (TICI) -2b, TICI-2c or TICI-3 recanalisation pattern after MT.
- mRS at 3 months. Its dichotomised assessment (mRS 0–2 indicating good functional recovery and 3–6 indicating death or dependency) is commonly used in acute stroke trials.
- Related to glycaemic control:
  - Peak interstitial glucose levels during the MT procedure.
  - Total time with interstitial glucose levels above 155 mg/dL from arrival in the emergency department to arterial recanalisation.
  - Time with interstitial glucose levels in the range of 110–154 mg/dL: (a) from arrival at the emergency department to recanalisation; (b) during the first 24 hours and (c) during hospitalisation.
  - Proportion of patients receiving insulin therapy for poststroke hyperglycaemia during the first 24 hours after onset of stroke symptoms.
  - Insulin therapy received in clinical practice (time, route and dose).
  - Number of CGMs with technical failures (missing or interrupted readings) or requiring replacement due to involuntary device removal or inadequate implantation.
  - Additional CGM device data: mean interstitial glucose level, coefficient of variation.
- Related to MT:
  - The number of MT passes (including aspiration and stent retriever passes).
  - Degree of recanalisation according to TICI classification on final angiogram.
  - Blood pressure in the emergency department and at the time of reperfusion.
- Related to stroke:
  - Infarct size at 24 hours (assessed by a neuroradiologist who will be unaware of the glucose monitoring data).
  - Haemorrhagic transformation at 24 hours (assessed by a neuroradiologist who will be unaware of the glucose monitoring data) is categorised into four types: (1) haemorrhagic infarction type 1 (HI-1)—small petechiae at the edges of the infarct area; (2) HI-2—confluent petechiae in the infarct area without mass effect; (3) parenchymal haematoma type 1 (HP-1)—haematoma occupying ≤30% of the infarct area with discrete mass effect and (4) parenchymal haematoma type 2 (HP-2)—haematoma occupying >30% of the infarct area with obvious mass effect.
  - Symptomatic haemorrhagic transformation is defined as CT/MRI evidence of any type of haemorrhage associated with neurological deterioration ≥4 points on the NIHSS scale from baseline or leading to death.
  - NIHSS score at 24 hours, at hospital discharge and at 3 months.
  - Neurological or systemic complications during follow-up. The following complications will be systematically assessed at each visit: coma, seizures, early neurological deterioration, cerebral oedema, recurrent stroke, acute coronary syndrome, pulmonary thromboembolism, respiratory infection, urinary tract infection, sepsis and local haematoma or infection at the insertion site of the subcutaneous glucose monitor. Any other complication reported or detected during follow-up will be also recorded.
  - Distribution of mRS scores at 90 days.
  - Mortality at 3 months.
- Biomarkers of ischaemia-reperfusion injury: miR-29b, miR-339, miR-15a, miR-100 and miR-424. Samples will be drawn at the time of reperfusion and 24 hours later.
- Demographics: age, sex, race, weight, height, diabetes, hypertension, dyslipidaemia, coronary disease, atrial fibrillation, metabolic syndrome, chronic kidney disease with an estimated Glomerular Filtration Rate (eGFR)<60 mL/min/1.73 m^2^, smoking and alcohol abuse.
- Previous drug therapy: antiplatelets, anticoagulants, antihypertensives, antidiabetics and lipid-lowering agents.
- Stroke data: date of symptom onset (or last asymptomatic event in patients with unknown stroke onset), aetiological subtype of stroke and treatment with intravenous thrombolysis.
- Vital signs and laboratory values on admission at the emergency department: temperature, blood pressure, heart rate, respiratory rate, blood glucose and glycated haemoglobin.
- Treatment times: time from stroke onset to hospital admission; time from hospital admission to start of intravenous thrombolysis (door-to-needle time); time from hospital admission to start of MT (door-to-femoral puncture time); time from hospital admission to reperfusion; time from femoral puncture to reperfusion.
- Neuroimaging data: Baseline Alberta Stroke Programme Early CT Score, baseline collateral flow and baseline perfusion.

Blood samples for miRNA analysis will be drawn from the antecubital vein at the time of reperfusion and 24 hours later. The miRNA analysis will be performed in the Neurological Sciences and Cerebrovascular Research Laboratory of IdiPAZ. Total RNA enriched in miRNA will be isolated from patient blood samples using the RNeasy mini kit (Qiagen, Germantown, Maryland, USA). cDNA will be generated from the extracted miRNA and amplified by a real-time PCR performed using a LightCycler system (Roche Diagnostics), RNA probes (Qiagen) and specific primers for each selected miRNA.

#### Data monitoring body

The data will be prospectively entered into a dedicated online database designed and maintained by the Clinical Trials Unit of La Paz University Hospital, which belongs to the Spanish Clinical Research Network. The entire process is governed by the principles of European biomedical research regulations, which ensure confidentiality. In accordance with European regulations and the International Conference of Harmonisation Good Clinical Practice Guidelines, the investigators and the institution will provide direct access to the authorised representatives of the ethics committees in the event that the original patient records are required for verification of the study data and procedures.

#### Sample size estimates

There have been no previous studies using CGM devices to assess the dynamics of glucose levels in patients treated with MT, which would allow a formal calculation of the sample size. Furthermore, the proportion of patients in whom technical failures may impede the recording of glycaemia at the time of reperfusion remains unknown. Using previous studies,^29 30^ if we consider a 15% loss to sensor failure or loss to follow-up, we can calculate, with an alpha risk of 0.05, a beta risk of 0.20 and an estimated difference in death-dependence of 30%, that it is recommended a sample size of 50 patients per group be used, taking into account that the death-dependence ratio in the control group might be 0.66 (according to GLIAS-II data). However, given that the actual percentage of possible technical failures of the sensor is unknown, we propose to perform an intermediate analysis and recalculate the sample size after the inclusion of 50 patients. The inclusion of 100 patients over a 2-year recruitment period is considered feasible, given that the number of MT procedures in our centre exceeds 100 per year. This sample size will allow us to investigate the differences in glycaemic levels at the time of reperfusion between patients with good or poor functional recovery at 3 months. After enrolling 100 patients, further power calculations will be performed to estimate the sample size for future studies. Recruitment began on 30 August 2023 and is ongoing.

### Statistical analysis

Statistical analysis will be conducted with the assistance of the Clinical Trials Unit of La Paz University Hospital. To analyse the continuous variables, the following information will be collected: the number of participants, mean, SD, median, minimum, maximum and 25th and 75th percentiles. For the categorical variables, the frequency distribution and 95% CI will be provided.

Multivariate logistic regression analyses will be performed to evaluate the effect of glucose levels at the time of reperfusion on functional recovery at 3 months, adjusting for other factors that show statistically significant differences in the univariate analysis. Significance will be assessed using the likelihood ratio.

## Discussion

This study represents a further step in the consolidated research into the prognostic impact of hyperglycaemia after stroke that began with the GLIAS study, the first prospective multicentre study to show that the glycaemic threshold associated with poor prognosis in acute stroke patients is 155 mg/dL.^13^ The GLIAS study also helped to highlight the significance of identifying and treating persistent hyperglycaemia.^40^ The GLIAS-II study (PS09/01781), funded by the Carlos III Health Institute (ISCIII), demonstrated the high incidence of non-response to conventional glycaemic control in patients with acute stroke and its association with poor outcomes.^18^ The GLIAS-III study (PI18/00991), currently finished and undergoing data analysis, is a multicentre, translational study analysing the impact of glycaemic variability evaluated by CGM on the progression of cerebral infarction in patients and in an animal model.^41^

The GLIAS-MT study aims to demonstrate that the impact of hyperglycaemia at the time of recanalisation is a critical modifiable prognostic factor, associated with greater brain damage, a different miRNA expression profile as a biomarker of damage, and poorer neurological and functional outcomes.

### Ethics and dissemination

#### Research ethics approval

The researchers will strictly adhere to the provisions of this protocol and will complete the case report forms. The study will be performed according to the recommendations for clinical studies and in the current Spanish and European legislation on clinical studies and patient data confidentiality. The study (protocol V1.1, dated 29 October 2021, code 6017) will follow the principles of Good Clinical Practice, has been approved by the Clinical Research Ethics Committee of La Paz University Hospital (Madrid, Spain) and is registered in ClinicalTrials.gov (NCT05871502). All modifications to the study protocol will be communicated by updating the registry at ClinicalTrials.gov.

#### Consent to participate

All participants will sign the informed consent document prior to any of the specific study procedures, which will comply with European standards of good clinical practice and data protection (EU 536/2014; EU 2016/679). In the case of patients with impaired consciousness, aphasia or neglect that could limit their understanding of the study objective and procedures, the signature of the patient’s guardian or legal representative will be acceptable. Reconsent from the patient will be obtained whenever possible if initial consent is provided by family members/representatives. The informed consent document is written in Spanish.

#### Confidentiality

Patient data will be anonymised prior to inclusion in the database. All participant information and the electronic database will remain in secure storage at the study centre during the study and up to 25 years after its finalisation.

#### Availability of data and materials

After the completion of the study, raw data will be deposited in an institutional repository, and the results will be published in an Open Access format.

#### Patient and public involvement

Patients and their advisors were not involved in the design, recruitment or conduct of this study.

#### Dissemination policy

The output of the study will include journal articles, conference presentations and community reports. None of the output will identify the participants.

## supplementary material

10.1136/bmjopen-2024-086745 online supplemental file 1
