# Supplementary material for: Glycaemia and ischaemia-reperfusion brain injury in patients with ischaemic stroke treated with mechanical thrombectomy (GLIAS-MT): an observational, unicentric, prospective study protocol
Source: BMJ Open. 2024 Aug 7;14(8):e086745. doi: 10.1136/bmjopen-2024-086745 (PMC11404171; doi:10.1136/bmjopen-2024-086745)
Supplement: online supplemental file 1 [file bmjopen-14-8-s001.pdf]

Supplemental Table 1. Treatment protocol for hyperglycaemia (>155 mg/dL)

<table>
<tr><td rowspan="10">During the first 24 hours: insulin should be administered via pump infusion, 100 International Unit (IU) of insulin in 100 ml of 0.9% saline, and the infusion rate adjusted according to the patient's hourly capillary glucose levels.
• If glucose levels above target >2 hours, increase infusion rate by 1IU/hour.
• If glucose levels >270mg/dL over 2 hours, increase infusion rate by 2IU/hour.
• If glucose levels >360mg/dL over 2 hours, increase infusion rate by 3 IU/hour.
The capillary blood glucose level should be monitored every hour until it remains within the target range for a period of four hours. Thereafter, the monitoring should be conducted every two hours.</td><td colspan="2">Intravenous (IV) insulin infusion regimen</td></tr>
<tr><td>Capillary glucose levels (mg/dL)</td><td>Insulin (IU/h)</td></tr>
<tr><td>155-179</td><td>1</td></tr>
<tr><td>180-209</td><td>1,5</td></tr>
<tr><td>210-239</td><td>2</td></tr>
<tr><td>240-269</td><td>3</td></tr>
<tr><td>270-299</td><td>4</td></tr>
<tr><td>300-329</td><td>5</td></tr>
<tr><td>330-359</td><td>6</td></tr>
<tr><td>>360</td><td>7</td></tr>
<tr><td colspan="3">Following a period of 24 hours and the initiation of oral/enteral nutrition:
1. If no iv insulin required during first 24 hours:
• Recent diagnosis of DM or a previous diagnosis of DM with antidiabetic drugs: 0.2 units of insulin glargine per kilogram of body weight per day, with rapid-acting insulin corrections.
• Previous diagnosis of DM with insulin therapy: 0.3-0.4 units of insulin glargine, with subsequent rapid-acting insulin corrections.
2. If iv insulin required during first 24 hours:
• Calculation of the total insulin dose with requirements during the previous 24 hours: 80% of the iv dose if requirements were ≤ 2IU/h, 50% if requirements were > 2IU/h.
• Overlap of iv insulin and subcutaneous (sc) insulin: maintain iv infusion until 2h after the first administration of rapid insulin sc or 4h if the insulin administered is long-acting.
• Insulin dose distribution: 50% basal insulin at 09am. 50% rapid-acting insulin divided into 4 doses at breakfast (20%), lunch (40%), snack (10%) and dinner (30%). Correction schedule with same analogue.</td></tr>
</table>
