# Supplementary material for: Glycaemia and ischaemia-reperfusion brain injury in patients with ischaemic stroke treated with mechanical thrombectomy (GLIAS-MT): an observational, unicentric, prospective study protocol
Source: BMJ Open. 2024 Aug 7;14(8):e086745. doi: 10.1136/bmjopen-2024-086745 (PMC11404171; doi:10.1136/bmjopen-2024-086745)
Supplement: Supplementary file 2 [file bmjopen-2024-086745.reviewer_comments.pdf]

PEER REVIEW HISTORY

BMJ Open publishes all reviews undertaken for accepted manuscripts. Reviewers are asked to complete a checklist review form (**http://bmjopen.bmj.com/site/about/resources/checklist.pdf**) and are provided with free text boxes to elaborate on their assessment. These free text comments are reproduced below.

ARTICLE DETAILS

**TITLE (PROVISIONAL)**	Glycaemia and ischaemia-reperfusion brain injury in patients with ischaemic stroke treated with mechanical thrombectomy (GLIAS-MT): an observational, unicentric, prospective study protocol
**AUTHORS**	Hervás, Carlos; Peirotén, Irene; González, Laura; Alonso de Leciñana, María; Alonso-López, Elisa; Casado, Laura; De Celis-Ruíz, Elena; Fernández Prieto, Andrés Francisco; Frutos, Remedios; Gallego-Ruiz, Rebeca; González Pérez de Villar, Noemí; Gutiérrez-Fernández, M.; Navia, Pedro; Otero-Ortega, Laura; Pozo-Novoa, Javier; Rigual, Ricardo; Rodríguez-Pardo, Jorge; Ruiz, Gerardo; Fuentes, Blanca

VERSION 1 – REVIEW

**REVIEWER**	Carcel, Cheryl<br>The George Institute for Global Health, Brain Health Program
**REVIEW RETURNED**	07-May-2024

**GENERAL COMMENTS**	1. Can the authors provide more details about miRNA, the mechanism as well how specific miRNAs were chosen.<br>2. Can the authors provide the protocol for treating hyperglycaemia?<br>3. How long will it take to insert the CGM device?<br>4. Where will the singlecentre study be performed in?<br>5. What is idiPaz and are you able to provide references?<br>Minor comment:<br>1. Please define miRNA at the first instance, page 7 line 19.

**REVIEWER**	Khamis, Assem<br>Hull York Medical School, Wolfson Palliative Care Research Centre
**REVIEW RETURNED**	08-May-2024

**GENERAL COMMENTS**	Peer Review: Glycaemia and ischaemia-reperfusion brain injury in patients with ischaemic stroke treated with mechanical thrombectomy. The GLIAS-MT study<br><br>This study protocol looks into the association between blood glucose levels and a range of health outcomes, mainly functional recovery. The introduction is well-written highlighting the gap in the literature about this topic. However, it would be great if the authors could include the reasoning behind choosing 155 mg/dL cut-off as part of the background before mentioning it as part of the study objectives. I was wondering why this cut-off was used until I found the answer in the discussion section citing Fuentes et al. study (ref no.34).

	In the Methods section:<br>Page 6, lines 49 & 55: endpoints are outcomes that should be listed as bullet points as under secondary endpoints, for example: 'the primary endpoint is stroke recovery. Secondary endpoints are: infarct volume, degree of haemorrhagic…., ….etc'. 'Evaluating the association' or 'exploring the influence' are parts of the statistical analysis which is detailed later.<br><br>Sample size calculation:<br>The authors could use the mortality percentage in a recent study to estimate the sample size for this study. For example: if the mortality rate is 25% as in SWIFT trial, the sample size would be 288, or 12.4% at 3 months as in the GLIAS study, the sample size would be 167. In addition, the authors should account for those who lost-to-follow up, withdrew or censored by 3-month period.<br><br>Statistical analysis:<br>I would say an opinion that the authors could ignore, they have a golden opportunity to explore the dose-response relationship between glucose levels (as a continuous variable) and functional recovery (as an ordinal variable) using multivariate ordinal/linear regression if possible. If not, I would recommend keeping glucose levels as a continuous variable in logistic regression.<br><br>Last, I would recommend against using the p-value cut-off for including variables in the multivariate model as it might exclude clinically important factors for future reference.

VERSION 1 – AUTHOR RESPONSE

Reviewer: 1

1. Can the authors provide more details about miRNA, the mechanism as well how specific miRNAs were chosen.

Thank you for proposing this course of action. A more detailed revision of the different miRNAs has been provided, along with an explanation of the rationale behind the selection of each one. Furthermore, miR100 has been included in the abstract. The new references have been added to the bibliography.

These miRNAs are of interest due to their functions, with previous studies demonstrating a role in cerebral ischaemia. In the context of ischemic stroke, miR-339, miR-15a, miR-424 and miR-100 have been associated with large vessel occlusion (33,34). MiR-339, which is overexpressed in cases of cerebral and myocardial ischaemia (35), is related to neuronal survival and suppression of apoptosis in ischaemic conditions (new reference 36). MiRNA 15 and 424 have been linked to an anti-angiogenic effect through VEGF inhibition (new reference 37). Additionally, miR-100 has been demonstrated to have an antiatherosclerotic effect (new reference 38). Furthermore, this miRNA exhibits varying levels in patients with cerebral infarction due to large vessel occlusion, suggesting a potential association with functional recovery (33). Finally, miR-29b has been demonstrated to attenuate ischemic injury by negatively regulating the p53-dependent apoptosis pathway, and could therefore be a potential target in diminishing cell injury in ischaemic stroke (new reference 39).

2. Can the authors provide the protocol for treating hyperglycaemia?

As previously stated, patients with glucose levels above 155 mg/dL will receive insulin at the discretion of the attending physician in accordance with the local protocol for managing hyperglycaemia in hospitalised patients and the guidelines for the management of hyperglycaemia in patients with stroke. This protocol is based on the GLIAS and GLIAS-II studies conducted in acute ischemic stroke patients in Spain, pointing out the threshold of 155 mg/dl as the one associated with poorer outcomes in our setting, and it is in alignment with the local multidisciplinary protocol for the management of in-hospital hyperglycaemia.

The aforementioned local protocols are included in a supplemental Table (Table S1. Treatment protocol for hyperglycaemia) for your convenience. To view the table properly, please click on the file attachment.

During the first 24 hours: insulin should be administered via pump infusion, 100 International Unit (IU) of insulin in 100 ml of 0.9% saline, and the infusion rate adjusted according to the patient's hourly capillary glucose levels.
• If glucose levels above target >2 hours, increase infusion rate by 1IU/hour.
• If glucose levels >270mg/dL over 2 hours, increase infusion rate by 2IU/hour.
• If glucose levels >360mg/dL over 2 hours, increase infusion rate by 3 IU/hour.
The capillary blood glucose level should be monitored every hour until it remains within the target range for a period of four hours. Thereafter, the monitoring should be conducted every two hours.
Intravenous (IV) insulin infusion regimen
Capillary glucose levels (mg/dL) Insulin (IU/h)
155-179 1
180-209 1,5
210-239 2
240-269 3
270-299 4
300-329 5
330-359 6
>360 7
Following a period of 24 hours and the initiation of oral/enteral nutrition:
1. If no iv insulin required during first 24 hours:
• Recent diagnosis of DM or a previous diagnosis of DM with antidiabetic drugs: 0.2 units of insulin glargine per kilogram of body weight per day, with rapid-acting insulin corrections.
• Previous diagnosis of DM with insulin therapy: 0.3-0.4 units of insulin glargine, with subsequent rapid-acting insulin corrections.
2. If iv insulin required during first 24 hours:
• Calculation of the total insulin dose with requirements during the previous 24 hours: 80% of the iv dose if requirements were ≤ 2IU/h, 50% if requirements were > 2IU/h.
• Overlap of iv insulin and subcutaneous (sc) insulin: maintain iv infusion until 2h after the first administration of rapid insulin sc or 4h if the insulin administered is long-acting.
• Insulin dose distribution: 50% basal insulin at 09am. 50% rapid-acting insulin divided into 4 doses at breakfast (20%), lunch (40%), snack (10%) and dinner (30%). Correction schedule with same analogue.

3. How long will it take to insert the CGM device?

The installation of the GCM device is a relatively straightforward process, requiring only a few seconds. It can be completed by both nurses and the medical team and is introduced beneath the skin with the aid of a simple applicator.

4. Where will the single centre study be performed in?

The study will be performed in La Paz University Hospital in Madrid. This is a comprehensive stroke center certified by the European Stroke Organisation.

5. What is idiPaz and are you able to provide references?

The Institute for Health Research of La Paz University Hospital (IdiPAZ) is a multidisciplinary and translational biomedical research centre specialised in basic, clinical, epidemiological and health services research. Further references may be found on the following website: https://idipaz.es/PaginaDinamica.aspx?IdPag=6&Lang=EN

6. Please define miRNA at the first instance, page 7 line 19.

Thank you for pointing this out. We have modified the manuscript accordingly.

Reviewer: 2

This study protocol looks into the association between blood glucose levels and a range of health outcomes, mainly functional recovery. The introduction is well-written highlighting the gap in the literature about this topic. However, it would be great if the authors could include the reasoning behind choosing 155 mg/dL cut-off as part of the background before mentioning it as part of the study objectives. I was wondering why this cut-off was used until I found the answer in the discussion section citing Fuentes et al. study (ref no.34).

We are grateful for your suggestion. The rationale behind this cutoff is explained in the aforementioned article. This cutoff is based on two studies, the GLIAS and the GLIAS II. We have added a shorter explanation and the reference to the GLIAS study as part of the background. We edited this part of the article in line with the above.

In the Methods section:
Page 6, lines 49 & 55: endpoints are outcomes that should be listed as bullet points as under secondary endpoints, for example: ‘the primary endpoint is stroke recovery. Secondary endpoints are: infarct volume, degree of haemorrhagic, etc. ‘Evaluating the association’ or ‘exploring the influence’ are parts of the statistical analysis which is detailed later.

We have implemented the suggested changes to the endpoints.

Primary endpoint:
• Stroke recovery at 3 months measured by the dichotomised mRS (0–2 indicating good functional recovery and 3–6 indicating death or dependency).

Secondary endpoints:
• Infarct volume at 24 h.
• Any degree of haemorrhagic transformation and symptomatic haemorrhagic transformation at 24 h.
• MicroRNA (miR-29b, miR-339, miR-15a, miR-100, and miR-424) expression profiles at the time of reperfusion and 24 h later.
• Neurological recovery at 24 h, using the National Institutes of Health Stroke Scale (NIHSS).
• Neurological and functional recovery at hospital discharge, using NIHSS and mRS.

Sample size calculation:
The authors could use the mortality percentage in a recent study to estimate the sample size for this

study. For example: if the mortality rate is 25% as in SWIFT trial, the sample size would be 288, or 12.4% at 3 months as in the GLIAS study, the sample size would be 167. In addition, the authors should account for those who lost-to-follow up, withdrew or censored by 3-month period.

Thank you for bringing this to our attention.

It is important to note that our study initially proposes a convenience sample size, given that there is a lack of complete knowledge regarding the relevant data for the sample size calculation. This study is one of the first to analyse the feasibility of sensors in patients with TM, and the percentage of patients in whom technical failures may prevent the recording of glycaemia at the time of reperfusion is currently unknown. A study of only 28 patients with ischaemic stroke treated with MT (reference 30) reports a failure rate of 13%. In contrast, another study reports excellent feasibility of using these sensors in patients with acute ischaemic stroke (new reference 29). Therefore, if we consider a 15% loss to sensor failure or loss to follow-up, using the Granmo calculator version 7.04 (https://www.imim.cat/media/upload/arxius/granmo/granmo_v704.html), we can calculate the following: with an alpha risk of 0.05, a beta risk of 0.20 and an estimated difference in death-dependence of 30%, it is recommended that a sample size of 50 patients per group be used, taking into account that the death-dependence ratio in the control group, according to GLIAS-II data, is 0.66. However, given that the actual percentage of possible technical failures of the sensor is unknown, we propose to perform an intermediate analysis and recalculate the sample size after inclusion of 50 patients. This will allow us to refine our estimates for the final size of our study.

We proceeded to edit the sample size section of the article in accordance with the agreed changes.

Statistical analysis:
I would say an opinion that the authors could ignore, they have a golden opportunity to explore the dose-response relationship between glucose levels (as a continuous variable) and functional recovery (as an ordinal variable) using multivariate ordinal/linear regression if possible. If not, I would recommend keeping glucose levels as a continuous variable in logistic regression. Last, I would recommend against using the p-value cut-off for including variables in the multivariate model as it might exclude clinically important factors for future reference.

Thank you for your valuable feedback. We will consider this and incorporate it into the statistical analysis.

VERSION 2 – REVIEW

**REVIEWER**	Carcel, Cheryl<br>The George Institute for Global Health, Brain Health Program
**REVIEW RETURNED**	18-Jun-2024

**GENERAL COMMENTS**	Thank you for addressing my comments

**REVIEWER**	Khamis, Assem<br>Hull York Medical School, Wolfson Palliative Care Research Centre
**REVIEW RETURNED**	02-Jul-2024

**GENERAL COMMENTS**	The authors addressed all the concerns. I have no further comments.
